# Prevalence of human papillomavirus genotypes and their variants in high risk West Africa women immigrants in South Italy

**DOI:** 10.1186/1750-9378-2-1

**Published:** 2007-01-03

**Authors:** Maria Lina Tornesello, Maria Luisa Duraturo, Luigi Buonaguro, Gabriele Vallefuoco, Roberto Piccoli, Stefano Palmieri, Franco M Buonaguro

**Affiliations:** 1 Viral Oncology and AIDS Reference Centre, National Cancer Institute, "Fond. Pascale", Naples, Italy; 2 Institute of Gynecology, University of Naples "Federico II", Naples, Italy; 3 Gynecology and Obstetric Unit, Pineta Grande Hospital, Caserta, Italy

## Abstract

**Background:**

The distribution of human papillomaviruses (HPVs) varies greatly across populations and HPV surveys have been performed in different geographical regions in order to apply appropriate vaccine strategies. Little information, however, exists regarding HPV genotypes distribution in immigrant women from countries at high incidence for cervical cancer. The aim of this study was to determine the spectrum of HPVs and their variants among HIV-positive and HIV-negative women immigrants in South Italy mainly from West Africa and with a history of prostitution.

**Results:**

Cervical cytological samples have been collected from 14 HIV-positive and 31 HIV-negative immigrants (38 out of 45 were born in Nigeria), attending a gynecological outpatient clinic in the Campania region. Human papillomaviruses were detected by broad spectrum consensus-primer-pairs MY09/MY11 and GP5+/GP6+-based polymerase chain reaction and characterized by nucleotide sequence analysis. Altogether, 42.2% (19/45) of samples were HPV positive with detection rates of 57.1% (8/14) in HIV-positive and 35.5% (11/31) in HIV-negative women. Among the twelve different viral genotypes identified, HPV33, 58, 70 and 81 were the prevalent genotypes with a frequency of 6.7% each, followed by HPV16, 35, 42, 54, 31, 52, 56 and 67, in descending order of prevalence. Sequence homology studies performed on the L1 amplified fragments of HPV16, 52 and 58 isolates allowed the identification of nucleotide changes distinctive of non-European variants.

**Conclusion:**

The overall HPV prevalence (42.2%) was high in this immigrant women group with the most common viral types other than HPV16 and 18, against which current vaccine strategies have been developed. The distribution of HPV genotypes and their variants in high-risk immigrants reflects that of their original countries. The surveillance of risk groups that may act as viral reservoirs of uncommon genotypes within different countries are necessary to determine the severity of HPV infection with the different viral types and to monitor a possible shift of prevalent strains following vaccination.

## Background

Human papillomaviruses (HPVs) are common pathogens associated with benign and malignant neoplasia of mucosal and cutaneous epithelia [1,2]. To date more than 100 HPV genotypes have been identified and at least 50 are known to infect the female anogenital tract [3,4]. Among these thirteen mucosotropic HPVs (types 16, 18, 31, 33, 35, 39, 45, 51, 52, 56, 58, 59, and 66) have been recently classified as class I carcinogens to human beings [5]. Several others types, however, need further studies being proposed as high risk viruses on the basis of 1) molecular phylogenetic relatedness to carcinogenic genotypes [3,6]; 2) epidemiological studies on the association with cervical cancer worldwide [7]; and 3) the *in vitro *biological properties [8]. The prevalence of HPV genotypes in cervical cytological samples varies greatly in different geographical regions and show a strong correlation with cervical cancer incidence [9-12]. The population-based HPV surveys coordinated by the International Agency for research on Cancer (IARC) reported that Nigeria had the highest prevalence of all HPV types and Europe the lowest, with nearly 20-fold variation between Nigeria (22.6%) and Spain (1.4%) [9,13,14]. The HPV type 16, although with different prevalence rates, is the most common viral type being present in 12.3%, 18.4%, 21.4% and 25.5% of HPV-positive cytological normal women from Sub-Saharan Africa (Nigeria), Asia, South America, and Europe, respectively [9]. Other mucosal HPVs are differently distributed in various geographical regions [9,15,16]. Factors that influence the prevalence rate of specific HPV types and eventually the outcome of cervical cancers are not clearly understood. However, it is well known that HPVs present well conserved genomic variants distinctive of geographical origin/population ethnicity [17]. The most extensively studied HPV16 variants cluster within five phylogenetic branches classified as European, Asian, Asian-American, African 1 and 2 variants [18-27], which differ in their biological properties and in their oncogenic potential [8,25,26,28]. Similar data have been described for HPV31, 33, 35, 45, 52 and 58, which can be grouped in several branches differing in geographic distribution, and in relative prevalence within different ethnical groups [29-31]. Genomic variants can be considered markers of specific HPV genomes and accordingly can be used in epidemiological and etiological studies to investigate transmission of HPV within and between populations [32].

The recent success of HPV16 and 18-based prophylactic vaccines in preventing persistent viral infections and HPV-associated cervical lesions is encouraging [33,34]. The vast heterogeneity of HPV infections, however, would require the development of vaccines targeting specific HPV types prevalent in a given population. Thus, extended analyses of type-specific HPVs in high risk populations will contribute to design appropriate large-scale screening tests and multivalent vaccine design strategies.

Several epidemiological studies have been performed among Italian women reporting the HPV type prevalence in cytological normal women, in low grade and high grade squamous intraepithelial lesions, in cervical cancer patients and in HIV-positive women [35-38]. All of them indicate that HPV16 is the most prevalent genotype in all analyzed patient's groups and that less common genotypes have an increased prevalence mainly in HIV-positive women [37].

No studies have been performed in Italy on the prevalence of HPV types in immigrant women with diverse risk profiles. The goal of the current study was to analyze HIV-positive and HIV-negative immigrant women, mainly from sub-Saharan Africa and with a history of prostitution, and to describe the prevalence of HPV genotypes and their variants in order to identify viral types that are spreading trough population mobility.

## Methods

### Study population and samples

This study enrolled 45 women attending the outpatient Unit of Obstetric and Gynecology Clinic of Pineta Grande Hospital from January through October 2004. This Hospital serves the highest number of long and short term immigrants in Campania region (Southern Italy). All women with sufficient Italian or English language skills, who voluntarily gave their consent to participate in the study, were interviewed using a structured questionnaire on reason for the visit, demographic characteristics and sexual behaviors. Fifteen women attended the clinic for prenatal care early in pregnancy, five for post-partum follow-up and 25 for routine gynecological care or various gynecological disorders including sexual transmitted diseases (STDs).

The cervical cell scrapings were collected with a cytobrush from the ecto- and endocervix of each woman. After spreading of cells on slides (Pap smears), the remaining cells were suspended in 1 ml of lysis buffer (10 mM Tris-HCl pH 7.6, 5 mM EDTA, 150 mM NaCl, 1% SDS) and stored at -20°C until analysis. Cytological evaluation of Pap smears, according to the 2001 Bethesda System, revealed two low-grade squamous intraepithelial lesions (LSIL) and 43 normal samples.

The study protocol was approved by the local ethical review board of authors' Institutions.

### HPV PCR amplifications

Genomic DNA was extracted from cervical-scraping cell lysates according to published procedures. In particular, samples were digested with Proteinase K (150 μg per ml at 60°C for 30 min) in 100 μl of lysis buffer (10 mM Tris-HCl pH 7.6, 5 mM EDTA, 150 mM NaCl, 1% SDS), followed by DNA purification by phenol and phenol-chloroform-isoamyl alcohol (25:24:1) extraction and Ethanol precipitation in 0.3 M Sodium Acetate (pH 4.6).

DNA quality test, performed by amplification with specific oligoprimers targeting a fragment of the exon 7 within the TP53 gene [28,39], and DNA quantity analysis, evaluated by spectro-photometric measurements, rendered all 45 samples suitable for viral DNA analysis.

HPV detection was performed by 1) single round PCR using the degenerated oligoprimers MY09/MY11 as previously described [40]; and 2) nested PCR methodology using as outer pair MY09/MY11 and as inner GP5+/GP6+ oligoprimers [41]. Both outer and inner PCR amplification reactions were performed in 50 μl reaction mixture containing 20 pmoles of each primer, 50 mM KCl, 3.75 mM MgCl2, 100 mM Tris-HCl pH 8.3, 0.1% Triton X-100, 50 mM of each dNTP and 1.8 units of thermostable AmpliTaq DNA polymerase (Applera, Courtaboeuf, France). For the first-amplification step of nested PCR, 5 μl of DNA (10 to 500 ng) was used as target DNA (outer reaction); in the second step PCR, 5 μl of the first step was used as input of amplified DNA (inner reaction). MY09/MY11 outer amplification reactions were performed in a Perkin-Elmer GeneAmp PCR System 9600 thermal cycler with the following steps: an initial 1-min denaturation at 94°C, followed by 32 cycles of 55°C for 1 min, 72°C for 2 min, 94°C for 30 s, and a final annealing at 55°C for 1 min with 5-min elongation at 72°C. GP5+/GP6+ inner amplification reactions were performed with an initial 1-min denaturation at 94°C followed by 40 cycles of 40°C for 1 min, 72°C for 1 min, 94°C for 1 min, and a final annealing of 40°C for 1 min followed by 4-min elongation at 72°C. A sample was considered HPV positive if one of the two amplification methods was positive, and negative if all tests were negative. Six plasmid clones containing HPV6, 11, 16, 18, 31 and 33 have been used as positive controls. To determine the sensitivity of both the single round MY09/MY11 PCR and the MY09/MY11-GP5+/GP6+ nested PCR, plasmid series of 10-fold dilutions (from 1000 to 1 copy/reaction) were tested by PCR. The detection limits of HPV16 and 31, were 1000 copies, with single round PCR, and 100 copies with nested PCR. The remaining four viral types were detected at 100 copies, with single round PCR, and 10 copies with nested PCR. These results are in agreement with the detection limits observed for mucosal HPVs following PCR amplification with the commonly used MY09/MY11 and GP5+/GP6+ oligoprimers [42]. Reaction mixtures without template DNA, included in every set of 5 clinical specimens, represented the negative controls.

### HPV DNA sequence analysis

HPV genotypes were identified by direct sequence analyses of single round MY09/MY11 amplified DNA and/or GP5+/GP6+ nested PCR amplified products obtained from each HPV-positive sample as previously described [38]. PCR products were extracted with phenol and chloroform-isoamyl alcohol and purified by precipitation at 37°C for 15 min in 1.25 M NaCl and 20% polyethylene glycol (PEG 6000) [43]. Purified DNA samples were subjected to direct nucleotide sequencing using a rapid method modified from Winship [44]. Briefly, DNA samples (30 ng to 100 ng) were denatured at 95°C, in presence of 10% DMSO, immediately cooled in liquid nitrogen and subsequently sequenced with the Sequenase 2.0 kit according to manufacturer's instructions (GE Healthcare) modified in the labeling step (3 min on ice). HPV type identification was performed by alignments of HPV sequences with those present in the GenBank database using the BLASTn software [45].

Two MY09/MY11 and three GP5+/GP6+ amplified DNA samples showing sequence patterns compatible with multiple infections have been subcloned in *Sma*I pBS-minus vector (Stratagene) and subjected to sequence analysis following procedures previously described [43].

HPV variants were searched by direct sequence analyses of MY09/MY11 amplified products (>400 bp long) obtained from each sample with a single HPV infection. The common sequence analysis limitation of low DNA amount obtained in some samples(< 50 ng) with single round MY09/MY11 PCR was circumvented by the use of AmpliCycle sequencing kit following the manufacturer's protocol of AmpliTaq DNA polymerase (Applera, Courtaboeuf, France), specific for 0.5 ng to 30 ng of PCR products. Two viral variants, amplified from samples containing multiple infections, have been characterized following subcloning into pBS-minus vector.

Phylogenetic analysis was performed by multiple sequence alignments of the 5' region of L1 genes from different HPV isolates with MegAlign program of the Lasergene software (DNASTAR Inc., 1994).

### Statistical analysis

The data were analyzed using χ^2 ^test and, where appropriate, Fisher's exact test to calculate all *P *values related to the differences between groups with Epi Info 6 Statistical Analysis System Software (6.04b, 1997, Centers for Disease Control and Prevention, USA). Differences were considered to be statistically significant when *P *values were less than 0.05.

## Results

### Characteristics of study population

In this study 45 randomly selected immigrant women (38 out of 45 were born in Nigeria) attending the gynecological outpatient unit of Pineta Grande Hospital in the Campania region have been enrolled from January through October 2004. Cervical cytological samples have been collected from all women consenting to the study after the administration of a standardized questionnaire regarding their life style and sexual activity. The selected demographic characteristics of the 14 HIV-positive and the 31 HIV-negative women have been reported in Table 1. The mean age was 29.48 (+/- 3.63) years for the HIV-positive and 28.85 (+/- 3.82) years for the HIV-negative women; 55.5% (25 out of 45) of the women reported past and/or current sexual risk behavior (i.e. history of prostitution).

**Table 1:** Characteristics of study population: HIV infection status in women belonging to different age strata and in accordance to history of prostitution

**Variable**	**Number of women**	**HIV-positive women (n = 14), n (%)**	**HIV-negative women (n = 31), n (%)**	***P *value**
Mean age [SD]	45	29.48 [± 3.63]	28.85 [± 3.82]	0.3088
				
Age				0.5616
<24	6	3 (50.0)	3 (50.0)	
25–30	14	4 (28.5)	10 (71.4)	
>30	25	7 (28.0)	18 (72.0)	
				
Country of Origin				0.0021
Nigeria	38	8 (21.0)	30 (78.9)	
Other*	7	6 (85.7)	1 (14.2)	
				
History of prostitution				0.4283
Yes	25	9 (36.0)	16 (64.0)	
No	20	5 (25.0)	15 (75.0)	
				
HPV status				0.1732
Positive	19	8 (42.1)	11 (57.8)	
Negative	26	6 (23.0)	20 (76.9)	

*Brazil (n = 1); Ethiopia (n = 1); Santo Domingo (n = 1); South Africa (n = 1); Ivory Coast (n = 1); Uruguay (n = 1).

### Prevalence of HPV infection and genotypes

All 45 samples have been analyzed by single round PCR using MY09/MY11 oligoprimers and samples negative or weakly positive for viral sequences have been subjected to nested PCR, using GP5+/GP6+ as inner oligoprimers. Overall, HPV sequences were amplified in 42.2% (19/45) of the samples with detection rates of 57.1% (8/14) in HIV-positive and 35.5% (11/31) in HIV-negative women (*P *= 0.1732). Fifteen subjects (60%) among the 25 reporting to have had sexual risk behavior tested positive for HPV sequences (*P *= 0.0165). Only 4 (20.0%) of the 20 women, without sexual risks behavior, were positive for HPV (Table 2). Distinct HPV genotypes have been identified by sequence analysis of MY09/MY11 or GP5+/GP6+ amplified products and by alignments of HPV sequences with those present in the GenBank database. Among the twelve different HPV types identified seven (HPV16, 31, 33, 35, 52, 56, 58) belong to the HPV virus group recently defined as class I carcinogenic to humans [5]. The HPV types 33 (6.7%), 58 (6.7%), 70 (6.7%) and 81 (6.7%) were the prevalent types as single infections followed by HPV16, 35, 42, 54, 31, 52, 56 and 67, in descending order of prevalence (Table 3). Multiple-type infections accounted for 26.3% of the HPV-infected women with no significant frequency difference between HIV-positive and HIV-negative subjects.

**Table 2:** HPV infection in women belonging to different age strata and in accordance to history of prostitution.

**Variable**	**Number of women**	**HPV-positive women (n = 19) n (%)**	**HPV-negative women (n = 19) n (%)**	***P *value**
Age				0.8843
<24	6	3 (50.0)	3 (50.0)	
25–30	14	6 (42.8)	8 (57.1)	
>30	25	10 (40.0)	15 (60.0)	
				
History of prostitution				0.0165*
Yes	25	15 (60.0)	10 (40.0)	
No	20	4 (20.0)	16 (80.0)	

*Yates corrected χ^2 ^test

**Table 3:** Prevalence of HPV genotypes in HIV-positive and HIV negative groups

**HPV genotype^a^**	**HIV-positive (n = 14), n (%)**	**HIV-negative (n = 31), n (%)**	**All Women (n = 45), n (%)**
HPV Positive	8 (57.1)	11 (35.5)	19 (42.2)
HPV Negative	6 (42.9)	20 (64.5)	26 (57.8)
			
Single infections			
**16**	**0 -**	**1 (3.2)**	**1 (2.2)**
**33**	**0 -**	**2 (6.4)**	**2 (4.4)**
**35**	**1 (7.1)**	**1 (3.2)**	**2 (4.4)**
**52**	**1 (7.1)**	**0 (3.2)**	**1 (2.2)**
**58**	**1 (7.1)**	**1 (3.2)**	**2 (4.4)**
67	1 (7.1)	0 -	1 (2.2)
70	2 (14.3)	0 -	2 (4.4)
54	0 -	1 (3.2)	1 (2.2)
81	1 (7.1)	1 (3.2)	2 (4.4)
	
Total single infections	7 (50.0)	7 (22.6)	14 (31.1)
			
Multiple infections^b^			
**56 (4) + 42 (2)**	**1 (7.1)**	**0 -**	**1 (2.2)**
**16 (5) + 42 (3)**	**0 -**	**1 (3.2)**	**1 (2.2)**
**31 (2) + 58 (5) + 35 (1)**	**0 -**	**1 (3.2)**	**1 (2.2)**
**33 (4) + 54 (2)**	**0 -**	**1 (3.2)**	**1 (2.2)**
70 (9) + 81 (1)	0 -	1 (3.2)	1 (2.2)
	
Total multiple infections	1 (7.1)	4 (12.9)	5 (11.1)

^a ^In bold case HPV genotypes defined by IARC working group as class I carcinogens for humans [5,50].

^b ^HPV genotypes present in multiple infections were characterized by subcloning of MY09/MY11 (two samples) or GP5+/GP6+ (three samples) PCR products and sequencing analysis. Number of clones analyzed for each HPV genotype are given in parenthesis.

### Identification and analysis of HPV variants

Sequence homology studies performed on the MY09/MY11 amplified regions of HPV16, 52 and 58 isolates allowed the identification of nucleotide changes distinctive of non-European viral variants. Table 4 presents a nucleotide comparison with the corresponding HPV reference sequences of six different viral isolates under study. The HPV16 sequence obtained from the DF-23 Nigerian sample is the only isolate 100% homologous to reference clone (European variant). The HPV16 sequence of the isolate DF-124, instead, shows seven nucleotide changes at positions 6695 (A to C), 6721 (G to A), 6853 (C to T), 6865 (C to T), 6970 (C to T), 6994 (G to A), and 7060 (G to T) which characterize HPV16 African 2 phylogenetic branch and have been previously identified in viral isolates from Africa [18,46], but not in the Italian population [28].

**Table 4:** Variants of Human papillomavirus types 16, 52 and 58 in immigrant women

**HPV16**	6 6 9 5	6 7 2 1	6 8 0 3	6 8 5 3	6 8 6 2	6 8 6 5	6 9 7 0	6 9 9 4	7 0 6 0	**Class**		**Country of origin**
**Reference (NC001526)**	A	G	A	C	T	C	C	G	G	E		Germany

**DF-23^a^**	-	-	-	-	-	-	-	-	-	E		Nigeria
**DF-124**	C	A	-	T	-	T	T	A	T	Af2		Nigeria
**Z-1194**	C	A	-	T	-	T	T	A	T	Af2		Zaire

**HPV52**	6 7 0 1	6 7 0 3	6 7 1 1	6 7 1 2	6 7 6 4	6 7 9 4	6 8 2 4	6 9 1 7	6 9 2 0	6 9 3 5	6 9 4 1	**Country of origin**

**Reference (X74481)**	T	A	A	G	T	A	C	C	T	T	A	United States

**DF-32**	-	C	G	A	-	-	-	A	G	A	G	Uruguay
**IS464 (U45923)**	-	C	G	A	-	-	-	A	G	A	G	Panama
**BR0258 (DQ057320)**	-	C	G	A	-	-	-	A	G	A	G	Brazil

**HK1151 (DQ057317)**	A	-	-	-	C	G	T	A	G	A	G	China

**HPV58**	6 6 4 1	6 6 8 8	6 6 9 2	6 6 9 6	6 6 9 7	6 7 1 1	6 7 9 8	6 8 2 2	6 8 2 7	6 8 2 8	7 0 1 6	**Country of origin**

**Reference (D90400)**	G	C	G	G	G	G	A	G	C	A	A	Japan

**DF-19**	-	A	-	-	-	-	G	A	A	G	-	Ethiopia
**Bsb-02 (AY101598)**	-	A	-	-	-	-	G	A	A	G	-	Brazil
**IS404 (U45928)**	-	-	-	-	-	-	G	A	A	G	-	Mali
												
**DF-03^a^**	-	-	A	A	-	A	G	A	A	G	-	Nigeria
**DF-09**	-	-	A	-	A	A	G	A	A	G	G	Nigeria
**IS417 (U45929)**	-	-	A	-	A	A	G	A	A	G	G	Mali
**Bsb-08 (AY098920)**	-	-	A	-	A	A	G	A	A	G	G	Brazil

Nucleotide positions, written vertically across the top, refer to nucleotide changes detected within MY09/MY11 L1 regions of HPV16, 52 and 58. Absence of genetic variations relative to the reference HPV type are marked with dashes, whereas presence of variant nucleotides are indicated by the nucleotide corresponding letter. Within each panel, the first column lists the variants identified in the present study (DF-09; DF-19; DF-23; DF-32; DF-124) and those previously described (Bsb-02 and Bsb-08 [44]; IS404, IS417 and IS464 [42]; BR0258 and HK1151 [26]; Z-1194 [59]) with the GeneBank accession number given in parenthesis.

^a^HPV variants identified in samples with multiple infections by subcloning of MY09/MY11 PCR-products and sequencing analyses.

The nucleotide sequence of one HPV52 viral isolate amplified from an Uruguayan sample, compared to the reference genome obtained from a North American women [28,47], showed seven nucleotide changes at nucleotide positions 6703 (A to C), 6711 (A to G), 6712 (G to A), 6917 (C to A), 6920 (T to G), 6935 T to A and 6941 (A to G), which have been previously reported in one sample from Panama [48] and one from Brazil [29]. Although the total number of HPV52 variant lineages and their geographical distribution has not yet been defined, our observation together with previous reports allow to postulate that the nucleotide changes common to isolates from Uruguay, Panama and Brazil could define a South American HPV52 lineage.

The sequence analysis of HPV58 isolates allowed the identification of four nucleotide changes at positions 6798 (G to A), 6822 (G to A), 6827 (C to A), 6828 (A to G), common to all three analyzed viral isolates and previously described in HPV58 samples from Mali and Brazil [48-50]; the nucleotide change at position 6688 (C to A) was present only in the Ethiopian sample DF-19 and has been previously reported in one HPV58 isolate from Brazil [50]; four nucleotide changes identified at positions 6692 (G to A) and 6711 (G to A), in the Nigerian samples DF-03 and DF-9, and at positions 6697 (G to A) and 7016 (A to G) identified only in the sample DF-09 have been previously reported in samples from Mali and Brazil [48,50]; the newly identified variations at position 6696 (G to A) has been observed only in the sample DF-03 and was present in all 5 plasmid clones selected for sequence analysis indicating that was not a PCR artifact. The pattern of MY09/MY11 HPV58 nucleotide changes previously identified in samples from Mali and Brazil, and also found in the African HPV58 isolates from this study, is compatible with the co-existence of at least two variant lineages in African and South American samples.

## Discussion

The prevalence of type specific HPVs and their variants has been analyzed in randomly selected immigrant women, mostly from Nigeria, living in Campania region. Viral infections have been detected by PCR using the MY09/MY11 oligoprimers alone or followed by nested PCR with GP5+/GP6+ primer pairs. Genotyping and variant characterization has been performed by direct sequencing analysis of PCR products. Samples which appeared to contain multiple infections for a mixed-sequence pattern were subcloned and nucleotide sequence has obtained from individual clones. The nested PCR method, performed with MY09/MY11, as outer primer pairs, and GP5+/GP6+, as inner primer pairs, has been previously shown to be significantly more sensitive (*P *< 0.001) for detection of all mucosal HPV genotypes and allows the identification of some HPV types, such as HPV30 and 70, not detected by either MY09/MY11 nor GP5+/GP6+ alone [38]. A total of twelve viral genotypes were identified including viral types (HPV16, 31, 33, 35, 52, 56 and 58) recently defined as class I oncogenic agents to human beings, and five low risk/undetermined risk viruses (HPV42, 54, 67, 70 and 81). The HPV prevalence of 35.5% observed among the HIV-negative immigrants, with an overall 42.2% independent from the HIV status, is higher than the HPV prevalence of 26.3% in Nigeria [13], and comparable to the prevalence of 40% in rural Mozambique [51], of 31% in Harare, Zimbabwe [16], and of 44% in Nairobi, Kenya [52]. As in most previous studies [13,16,51,52], also multiple HPV infections have been found in a substantial fraction of HPV positive women (11.1%).

Moreover, 31.1% of the women analyzed in this study were HIV positive, and this value is much higher than HIV prevalence of 22.6% among female sex workers in Ibadan (Nigeria), as reported by the UNAIDS HIV Surveillance [53].

Several reports pointed out the possibility of certain HPV types being more common in sub-Saharan African women than elsewhere. HPV35, for instance, was slightly more common than HPV16 in Mozambique both in women with normal cytology and in those with high-grade squamous intraepithelial lesions or worse [51]. HPV52 was found slightly more frequently than HPV16 or HPV35 in Kenya [52] and in colposcopically normal women in Zimbabwe [16]. In Nigeria HPV16 and 35 were the most common high risk types detected in normal and dysplatic lesions, followed by HPV31, 58, and 56 [13]. In the present study when the frequency of HPV types both in single and multiple infections were combined, HPV33, 58, 70 and 81 represented each 6.7% of infections followed by HPV16, 35, 42 and 54 (4.4% each type). The HPV70, which was epidemiological classified as a low risk type on the basis of its detection in two cases and two controls in a large study performed in nine countries [7], has been previously reported as single infection in cervical carcinomas from Asia (China) [54], Japan [55] and Thailand [56]), South America (Mexico [57], and Peru [58]); and in high grade squamous intraepithelial lesions in Italy [38]. Recently it has been shown by *in vitro *studies that HPV70 together with 53 and 82, which are phylogenetically related to high risk viruses, are indistinguishable with respect to their ability to degrade p53 and to immortalize keratinocytes, suggesting the need to monitor this HPV types in more extended epidemiological studies [8].

Phylogenetic studies of HPV16, 18, and the less prevalent HPV types 31, 33, 35, 52, 58, and 67, obtained from different geographical regions, showed that specific variants can be identified in different ethnic groups from different geographical regions [29,48,50].

In this study only one HPV isolate out of six, analyzed within the MY09/MY11 region, presented homology with the reference sequence of the corresponding genotype. A second HPV16 isolate obtained from a Nigerian woman showed nucleotide signatures distinctive of HPV16 African 2 lineage, which was never detected in the Italian population. The single HPV52 isolate obtained from an Uruguayan woman, showed nucleotide changes specific of viral variants previously identified in samples from Panama and Brazil [28,29,48,48]. Furthermore, three HPV58 isolates, one obtained from an Ethiopian and two from Nigerian women, were homologous to two distinct viral variants previously identified in Mali and Brazil [48]. The presence of similar multiple HPV variants in African and South-American geographical regions can be explained by the extensive African immigration to Brazil during the colonization of the American continent.

Although this study has the limitation to include a relatively low number of women, due to the low participation rate, it clearly shows that migrant women carries the viral types and their variants present in their original countries, with possible implications in the spreading of uncommon HPV genotypes in the resident populations.

## Conclusion

In conclusion, the immigrant women analyzed in this study, mainly of African origin and with sexual risk behavior, have a high HPV prevalence with the most common viral types other than HPV16 and 18, against which current vaccine strategies have been developed. Wide population based epidemiological studies, including the surveillance of risk groups that may act as niches of uncommon viral types and their variants, are necessary, particularly in countries like Italy with high immigration rates, to monitor eventual spreading of uncommon genotypes for vaccine considerations.

## Competing interests

The author(s) declare that they have no competing interests.

## Authors' contributions

FMB, MLT and SP were responsible for the overall planning and coordination of the immigrant women community based study.  LB was involved in phylogenetic analyses. GV and RP were involved in the patients enrollment and cervical sample collection. MLD was responsible for specimen processing and HPV-DNA analysis. MLT and FMB compiled and finalized the manuscript. All authors read and approved the final manuscript.

## References

[B1] zur Hausen H (2000). Papillomaviruses causing cancer: evasion from host-cell control in early events in carcinogenesis. J Natl Cancer Inst.

[B2] IARC (1995). IARC monographs on the evaluation of carcinogenic risks to humans Human Papillomaviruses.

[B3] de Villiers EM, Fauquet C, Broker TR, Bernard HU, zur Hausen H (2004). Classification of papillomaviruses. Virology.

[B4] de Villiers EM, Whitley C, Gunst K (2005). Identification of new papillomavirus types. Methods Mol Med.

[B5] Cogliano V, Baan R, Straif K, Grosse Y, Secretan B, El GF (2005). Carcinogenicity of human papillomaviruses. Lancet Oncol.

[B6] Bravo IG, Alonso A (2004). Mucosal human papillomaviruses encode four different E5 proteins whose chemistry and phylogeny correlate with malignant or benign growth. J Virol.

[B7] Munoz N, Bosch FX, de Sanjose S, Herrero R, Castellsague X, Shah KV, Snijders PJ, Meijer CJ (2003). Epidemiologic classification of human papillomavirus types associated with cervical cancer. N Engl J Med.

[B8] Hiller T, Poppelreuther S, Stubenrauch F, Iftner T (2006). Comparative analysis of 19 genital human papillomavirus types with regard to p53 degradation, immortalization, phylogeny, and epidemiologic risk classification. Cancer Epidemiol Biomarkers Prev.

[B9] Clifford GM, Gallus S, Herrero R, Munoz N, Snijders PJ, Vaccarella S, Anh PT, Ferreccio C, Hieu NT, Matos E, Molano M, Rajkumar R, Ronco G, de SS, Shin HR, Sukvirach S, Thomas JO, Tunsakul S, Meijer CJ, Franceschi S (2005). Worldwide distribution of human papillomavirus types in cytologically normal women in the International Agency for Research on Cancer HPV prevalence surveys: a pooled analysis. Lancet.

[B10] Clifford GM, Rana RK, Franceschi S, Smith JS, Gough G, Pimenta JM (2005). Human papillomavirus genotype distribution in low-grade cervical lesions: comparison by geographic region and with cervical cancer. Cancer Epidemiol Biomarkers Prev.

[B11] Clifford GM, Smith JS, Aguado T, Franceschi S (2003). Comparison of HPV type distribution in high-grade cervical lesions and cervical cancer: a meta-analysis. Br J Cancer.

[B12] Clifford GM, Smith JS, Plummer M, Munoz N, Franceschi S (2003). Human papillomavirus types in invasive cervical cancer worldwide: a meta-analysis. Br J Cancer.

[B13] Thomas JO, Herrero R, Omigbodun AA, Ojemakinde K, Ajayi IO, Fawole A, Oladepo O, Smith JS, Arslan A, Munoz N, Snijders PJ, Meijer CJ, Franceschi S (2004). Prevalence of papillomavirus infection in women in Ibadan, Nigeria: a population-based study. Br J Cancer.

[B14] de SS, Almirall R, Lloveras B, Font R, Diaz M, Munoz N, Catala I, Meijer CJ, Snijders PJ, Herrero R, Bosch FX (2003). Cervical human papillomavirus infection in the female population in Barcelona, Spain. Sex Transm Dis.

[B15] Xi LF, Toure P, Critchlow CW, Hawes SE, Dembele B, Sow PS, Kiviat NB (2003). Prevalence of specific types of human papillomavirus and cervical squamous intraepithelial lesions in consecutive, previously unscreened, West-African women over 35 years of age. Int J Cancer.

[B16] Gravitt PE, Kamath AM, Gaffikin L, Chirenje ZM, Womack S, Shah KV (2002). Human papillomavirus genotype prevalence in high-grade squamous intraepithelial lesions and colposcopically normal women from Zimbabwe. Int J Cancer.

[B17] Xi LF, Kiviat NB, Hildesheim A, Galloway DA, Wheeler CM, Ho J, Koutsky LA (2006). Human papillomavirus type 16 and 18 variants: race-related distribution and persistence. J Natl Cancer Inst.

[B18] Ho L, Chan SY, Chow V, Chong T, Tay SK, Villa LL, Bernard HU (1991). Sequence variants of human papillomavirus type 16 in clinical samples permit verification and extension of epidemiological studies and construction of a phylogenetic tree. J Clin Microbiol.

[B19] Chan SY, Ho L, Ong CK, Chow V, Drescher B, Durst M, ter Meulen J, Villa L, Luande J, Mgaya HN (1992). Molecular variants of human papillomavirus type 16 from four continents suggest ancient pandemic spread of the virus and its coevolution with humankind. J Virol.

[B20] Xi LF, Demers GW, Koutsky LA, Kiviat NB, Kuypers J, Watts DH, Holmes KK, Galloway DA (1995). Analysis of human papillomavirus type 16 variants indicates establishment of persistent infection. J Infect Dis.

[B21] Song YS, Kee SH, Kim JW, Park NH, Kang SB, Chang WH, Lee HP (1997). Major sequence variants in E7 gene of human papillomavirus type 16 from cervical cancerous and noncancerous lesions of Korean women. Gynecol Oncol.

[B22] Zehbe I, Wilander E, Delius H, Tommasino M (1998). Human papillomavirus 16 E6 variants are more prevalent in invasive cervical carcinoma than the prototype. Cancer Res.

[B23] Buonaguro FM, Tornesello ML, Salatiello I, Okong P, Buonaguro L, Beth-Giraldo E, Biryahwaho B, Sempala SD, Giraldo G (2000). The Uganda study on HPV variants and genital cancers. J Clin Virol.

[B24] Villa LL, Sichero L, Rahal P, Caballero O, Ferenczy A, Rohan T, Franco EL (2000). Molecular variants of human papillomavirus types 16 and 18 preferentially associated with cervical neoplasia. J Gen Virol.

[B25] Berumen J, Ordonez RM, Lazcano E, Salmeron J, Galvan SC, Estrada RA, Yunes E, Garcia-Carranca A, Gonzalez-Lira G, Madrigal-de la Campa A (2001). Asian-American variants of human papillomavirus 16 and risk for cervical cancer: a case-control study. J Natl Cancer Inst.

[B26] Hildesheim A, Schiffman M, Bromley C, Wacholder S, Herrero R, Rodriguez A, Bratti MC, Sherman ME, Scarpidis U, Lin QQ, Terai M, Bromley RL, Buetow K, Apple RJ, Burk RD (2001). Human papillomavirus type 16 variants and risk of cervical cancer. J Natl Cancer Inst.

[B27] Burk RD, Terai M, Gravitt PE, Brinton LA, Kurman RJ, Barnes WA, Greenberg MD, Hadjimichael OC, Fu L, McGowan L, Mortel R, Schwartz PE, Hildesheim A (2003). Distribution of human papillomavirus types 16 and 18 variants in squamous cell carcinomas and adenocarcinomas of the cervix. Cancer Res.

[B28] Tornesello ML, Duraturo ML, Salatiello I, Buonaguro L, Losito S, Botti G, Stellato G, Greggi S, Piccoli R, Pilotti S, Stefanon B, De PG, Franceschi S, Buonaguro FM (2004). Analysis of human papillomavirus type-16 variants in Italian women with cervical intraepithelial neoplasia and cervical cancer. J Med Virol.

[B29] Calleja-Macias IE, Villa LL, Prado JC, Kalantari M, Allan B, Williamson AL, Chung LP, Collins RJ, Zuna RE, Dunn ST, Chu TY, Cubie HA, Cuschieri K, von Knebel-Doeberitz M, Martins CR, Sanchez GI, Bosch FX, Munoz N, Bernard HU (2005). Worldwide genomic diversity of the high-risk human papillomavirus types 31, 35, 52, and 58, four close relatives of human papillomavirus type 16. J Virol.

[B30] Prado JC, Calleja-Macias IE, Bernard HU, Kalantari M, Macay SA, Allan B, Williamson AL, Chung LP, Collins RJ, Zuna RE, Dunn ST, Ortiz-Lopez R, Barrera-Saldana HA, Cubie HA, Cuschieri K, von Knebel-Doeberitz M, Sanchez GI, Bosch FX, Villa LL (2005). Worldwide genomic diversity of the human papillomaviruses-53, 56, and 66, a group of high-risk HPVs unrelated to HPV-16 and HPV-18. Virology.

[B31] Calleja-Macias IE, Kalantari M, Huh J, Ortiz-Lopez R, Rojas-Martinez A, Gonzalez-Guerrero JF, Williamson AL, Hagmar B, Wiley DJ, Villarreal L, Bernard HU, Barrera-Saldana HA (2004). Genomic diversity of human papillomavirus-16, 18, 31, and 35 isolates in a Mexican population and relationship to European, African, and Native American variants. Virology.

[B32] Bernard HU, Calleja-Macias IE, Dunn ST (2006). Genome variation of human papillomavirus types: phylogenetic and medical implications. Int J Cancer.

[B33] Harper DM, Franco EL, Wheeler CM, Moscicki AB, Romanowski B, Roteli-Martins CM, Jenkins D, Schuind A, Costa Clemens SA, Dubin G (2006). Sustained efficacy up to 4.5 years of a bivalent L1 virus-like particle vaccine against human papillomavirus types 16 and 18: follow-up from a randomised control trial. Lancet.

[B34] Villa LL, Costa RL, Petta CA, Andrade RP, Ault KA, Giuliano AR, Wheeler CM, Koutsky LA, Malm C, Lehtinen M, Skjeldestad FE, Olsson SE, Steinwall M, Brown DR, Kurman RJ, Ronnett BM, Stoler MH, Ferenczy A, Harper DM, Tamms GM, Yu J, Lupinacci L, Railkar R, Taddeo FJ, Jansen KU, Esser MT, Sings HL, Saah AJ, Barr E (2005). Prophylactic quadrivalent human papillomavirus (types 6, 11, 16, and 18) L1 virus-like particle vaccine in young women: a randomised double-blind placebo-controlled multicentre phase II efficacy trial. Lancet Oncol.

[B35] Ronco G, Ghisetti V, Segnan N, Snijders PJ, Gillio-Tos A, Meijer CJ, Merletti F, Franceschi S (2005). Prevalence of human papillomavirus infection in women in Turin, Italy. Eur J Cancer.

[B36] De Francesco MA, Gargiulo F, Schreiber C, Ciravolo G, Salinaro F, Manca N (2005). Detection and genotyping of human papillomavirus in cervical samples from Italian patients. J Med Virol.

[B37] Cappiello G, Garbuglia AR, Salvi R, Rezza G, Giuliani M, Pezzotti P, Suligoi B, Branca M, Migliore G, Formigoni Pomponi D, D'Ubaldo C, Ippolito G, Giacomini G, Benedetto A (1997). HIV infection increases the risk of squamous intra-epithelial lesions in women with HPV infection: an analysis of HPV genotypes. DIANAIDS Collaborative Study Group. Int J Cancer.

[B38] Tornesello ML, Duraturo ML, Botti G, Greggi S, Piccoli R, De Palo G, Montella M, Buonaguro L, Buonaguro FM (2006). Prevalence of alpha-papillomavirus genotypes in cervical intraepithelial neoplasia and cervical cancer in the Italian population. J Med Virol.

[B39] Buonaguro FM, Tornesello ML, Beth-Giraldo E, Hatzakis A, Mueller N, Downing R, Biryamwaho B, Sempala SD, Giraldo G (1996). Herpesvirus-like DNA sequences detected in endemic, classic, iatrogenic and epidemic Kaposi's sarcoma (KS) biopsies. Int J Cancer.

[B40] Resnick RM, Cornelissen MT, Wright DK, Eichinger GH, Fox HS, ter Schegget J, Manos MM (1990). Detection and typing of human papillomavirus in archival cervical cancer specimens by DNA amplification with consensus primers. J Natl Cancer Inst.

[B41] de Roda Husman AM, Walboomers JM, van den Brule AJ, Meijer CJ, Snijders PJ (1995). The use of general primers GP5 and GP6 elongated at their 3' ends with adjacent highly conserved sequences improves human papillomavirus detection by PCR. J Gen Virol.

[B42] Quint WG, Pagliusi SR, Lelie N, de Villiers EM, Wheeler CM (2006). Results of the first World Health Organization international collaborative study of detection of human papillomavirus DNA. J Clin Microbiol.

[B43] Tornesello ML, Buonaguro FM, Buonaguro L, Salatiello I, Beth-Giraldo E, Giraldo G (2000). Identification and functional analysis of sequence rearrangements in the long control region of human papillomavirus type 16 Af-1 variants isolated from Ugandan penile carcinomas. J Gen Virol.

[B44] Winship PR (1989). An improved method for directly sequencing PCR amplified material using dimethyl sulphoxide. Nucleic acids Res.

[B45] Nucleotide-nucleotide BLAST. http://www.ncbi.nlm.nih.gov/blast/.

[B46] Chen Z, Terai M, Fu L, Herrero R, DeSalle R, Burk RD (2005). Diversifying selection in human papillomavirus type 16 lineages based on complete genome analyses. J Virol.

[B47] Shimoda K, Lorincz AT, Temple GF, Lancaster WD (1988). Human papillomavirus type 52: a new virus associated with cervical neoplasia. J Gen Virol.

[B48] Stewart AC, Eriksson AM, Manos MM, Munoz N, Bosch FX, Peto J, Wheeler CM (1996). Intratype variation in 12 human papillomavirus types: a worldwide perspective. J Virol.

[B49] Veras VS, Cerqueira DM, Martins CR (2005). L1 sequence of a new human papillomavirus type-58 variant associated with cervical intraepithelial neoplasia. Braz J Med Biol Res.

[B50] Cerqueira DM, Camara GN, da Cruz MR, Silva EO, Brigido MM, Carvalho LG, Martins CR (2003). Variants of human papillomavirus types 53, 58 and 66 identified in Central Brazil. Virus Genes.

[B51] Castellsague X, Menendez C, Loscertales MP, Kornegay JR, dos Santos F, Gomez-Olive FX, Lloveras B, Abarca N, Vaz N, Barreto A, Bosch FX, Alonso P (2001). Human papillomavirus genotypes in rural Mozambique. Lancet.

[B52] De Vuyst H, Steyaert S, Van RL, Claeys P, Muchiri L, Sitati S, Vansteelandt S, Quint W, Kleter B, Van ME, Temmerman M (2003). Distribution of human papillomavirus in a family planning population in nairobi, kenya. Sex Transm Dis.

[B53] (2007). UNAIDS HIV Surveillance. http://www.unaids.org.

[B54] Lin QQ, Yu SZ, Qu W, Cruz Y, Burk RD (1998). Human papillomavirus types 52 and 58. Int J Cancer.

[B55] Nakagawa S, Yoshikawa H, Onda T, Kawana T, Iwamoto A, Taketani Y (1996). Type of human papillomavirus is related to clinical features of cervical carcinoma. Cancer.

[B56] Chichareon S, Herrero R, Munoz N, Bosch FX, Jacobs MV, Deacon J, Santamaria M, Chongsuvivatwong V, Meijer CJ, Walboomers JM (1998). Risk factors for cervical cancer in Thailand: a case-control study. J Natl Cancer Inst.

[B57] Torroella-Kouri M, Morsberger S, Carrillo A, Mohar A, Meneses A, Ibarra M, Daniel RW, Ghaffari AM, Solorza G, Shah KV (1998). HPV prevalence among Mexican women with neoplastic and normal cervixes. Gynecol Oncol.

[B58] Santos C, Munoz N, Klug S, Almonte M, Guerrero I, Alvarez M, Velarde C, Galdos O, Castillo M, Walboomers J, Meijer C, Caceres E (2001). HPV types and cofactors causing cervical cancer in Peru. Br J Cancer.

[B59] Roden RB, Armstrong A, Haderer P, Christensen ND, Hubbert NL, Lowy DR, Schiller JT, Kirnbauer R (1997). Characterization of a human papillomavirus type 16 variant- dependent neutralizing epitope. J Virol.

